# MicroRNAs in Ewing Sarcoma

**DOI:** 10.3389/fonc.2013.00065

**Published:** 2013-03-28

**Authors:** Layne Dylla, Colin Moore, Paul Jedlicka

**Affiliations:** ^1^Medical Scientist Training Program, University of Colorado DenverDenver, CO, USA; ^2^Cancer Biology Graduate Program, University of Colorado DenverDenver, CO, USA; ^3^Anschutz Medical Campus, University of Colorado DenverDenver, CO, USA; ^4^Center for Cancer and Blood Disorders, University of Colorado DenverAurora, CO, USA; ^5^Departments of Pediatrics, University of Colorado DenverDenver, CO, USA; ^6^Children’s Hospital ColoradoAurora, CO, USA; ^7^Department of Pathology, University of Colorado DenverDenver, CO, USA

**Keywords:** microRNAs, sarcoma, Ewing sarcoma, pathogenesis, prognosis, therapy

## Abstract

MicroRNAs (miRs) have emerged recently as important regulators of gene expression in the cell. Frequently dysregulated in cancer, miRs have shed new light on molecular mechanisms of oncogenesis, and have generated substantial interest as biomarkers, and novel therapeutic agents and targets. Recently, a number of studies have examined miR biology in Ewing sarcoma. Findings indicate that alterations in miR expression in Ewing Sarcoma are widespread, involve both EWS/Ets oncogenic fusion-dependent and independent mechanisms, and contribute to malignant phenotypes. miRs with prognostic potential have been identified, and several preclinical studies suggest that miR manipulation could be therapeutically useful in this aggressive disease. These and future studies of miR biology stand to expand our understanding of Ewing sarcoma pathogenesis, and may identify new biomarkers and treatment options.

## Introduction

MicroRNAs (miRs) represent a recently discovered novel class of cellular bioactive molecules with important functions in the regulation of gene expression in normal physiology and disease (Ghildiyal and Zamore, 2009). MiRs are short (20–30 nt) single-stranded RNA molecules that bind to protein-coding messenger RNA (mRNA) molecules, predominantly in the 3′ untranslated region (UTR) (Ghildiyal and Zamore, 2009). This binding results in decreased synthesis of the coded protein, by a number of mechanisms including increased mRNA degradation and inhibition of translation (Ghildiyal and Zamore, 2009). The binding is sequence-specific, but involves a limited (∼6–8) nt match (Bartel, 2009). Thus, individual miRs have many possible mRNA targets, while, as a group, miRs contribute to the control of expression of much of the genome.

MicroRNAs are derived from hairpin-shaped, double-stranded precursors (pre-miRs) by the action of a protein complex containing the Dicer gene product (Davis and Hata, 2009; Kim et al., 2009; Winter et al., 2009). Most of these precursors are in turn derived from longer primary transcripts (pri-miRs) by the action of the microprocessor complex containing the proteins Drosha and DGCR8 (Davis and Hata, 2009; Kim et al., 2009; Winter et al., 2009). Although some miRs are embedded in protein-coding genes and co-regulated with the parent mRNA, approximately one-half are derived from independent, non-protein-coding transcripts under control of RNA Polymerase II-driven promoters (Davis and Hata, 2009; Kim et al., 2009; Winter et al., 2009). The expression of such miRs is subject to the same mechanisms of promoter regulation as protein-coding genes, including the action of specific transcription factors. Relatively little is currently known about the precise mechanisms controlling miR expression under conditions of normal homeostasis and in disease.

In cancer, miRs function as context-dependent tumor suppressors or oncogenes, capable, through their molecular function as regulators of gene expression, of modifying all aspects of tumorigenesis, including tumor cell proliferation/apoptosis, invasion/metastasis, stem-like properties, and angiogenesis (Sotiropoulou et al., 2009; Visone and Croce, 2009). Importantly, miRs represent promising new therapeutic agents or/and targets, a concept borne out in preclinical studies (Weidhaas et al., 2007; Trang et al., 2008; Wang and Wu, 2009; Kasinski and Slack, 2011; Nana-Sinkam and Croce, 2011). Such studies have shown that administration of chemical mimics of tumor suppressive miR, or chemical antagonists of pro-tumorigenic miRs, can have potent effects on tumor growth or/and dissemination in disease animal models. Examples of successful preclinical therapeutic trials in pediatric cancers include miR-380-5p replacement in neuroblastoma (Swarbrick et al., 2010), and miR replacement/anti-miR combination therapy, involving miR-100 and miR-371 clusters, in hepatoblastoma (Cairo et al., 2010). Thus, miR manipulation *in vivo* can affect disease phenotypes. Some miR-based therapies have already moved ahead to clinical trials, including blockade of miR-122 for chronic viral hepatitis (Cho, 2010; Wahid et al., 2010).

Recently, a number of studies have examined the biology of miRs in Ewing Sarcoma. The purpose of this minireview is to summarize the findings of these studies, and discuss insights that they have provided into disease pathogenesis, and potential options for improved disease subclassification and therapy.

## MicroRNAs in EWS/Fli1-Driven Oncogenesis

The pathogenesis of the vast majority of Ewing Sarcomas is driven by EWS/Ets fusion oncoproteins, which arise from recurrent chromosomal translocations and are necessary for tumorigenesis (Janknecht, 2005; Jedlicka, 2010; Toomey et al., 2010). EWS/Ets fusions, with EWS/Fli1 being the most common, consist of the amino terminus of the EWS gene and the carboxy terminus, including the DNA-binding domain, of an Ets transcription factor gene. Transcriptional activity, including both activation and repression, is central to EWS/Ets oncogenic action (Toomey et al., 2010; Sankar et al., 2012). Additionally, EWS/Ets downstream genes include other transcriptional regulators (Toomey et al., 2010). Furthermore, the EWS protein may play a role in miR processing (Gregory et al., 2004; Sohn et al., 2012). Since transcription and processing represent important mechanisms of control of miR levels in the cell (Davis and Hata, 2009; Kim et al., 2009; Winter et al., 2009), it was reasonable to postulate that EWS/Ets fusions affect the expression of miRs in Ewing Sarcoma, and that consequent alterations in miR levels contribute to the execution of the EWS/Ets-driven oncogenic program. Identification and characterization of EWS/Ets-regulated miRs was undertaken by several groups.

Ban et al. (2011) used transient siRNA-mediated knock-down to deplete EWS/Fli1 in five different Ewing Sarcoma cell lines. They then compared miR levels between control and EWS/Fli1-depleted cells, and between five Ewing Sarcoma patient tumors and mesenchymal stem cells (MSCs, the presumed cells of Ewing Sarcoma origin) from six different individuals, using a multiplexed RT-qPCR platform. This approach identified 15 upregulated and 14 downregulated miRs across all comparison groups. MiR-145 was the miR most consistently changed, namely reduced upon EWS/Fli1 depletion, and underexpressed in Ewing Sarcoma relative to MSCs. In support of a tumor suppressive role, the authors showed that miR-145 replacement results in inhibition of anchorage-independent growth of Ewing Sarcoma cells. Further, they showed direct repression of EWS/Fli1 by miR-145, suggesting the existence of a miR-mediated positive feedback loop for augmenting EWS/Fli1 protein levels in the cell. A similar feedback loop has been identified by Riggi et al. (2010), as discussed in more detail below. A subsequent study has shown that miR-708, another EWS/Fli1-downregulated miR identified in the studies of Ban et al., modulates chemotherapy responsiveness in Ewing Sarcoma (Robin et al., 2012).

McKinsey et al. (2011) took the approach of stably knocking down EWS/Fli1 in Ewing Sarcoma A673 cells, using lentivirally delivered shRNAs, and probing for changes in miR levels using a miR microarray platform. This approach identified 29 upregulated, and 31 downregulated miRs upon EWS/Fli1 depletion. Focusing on a group of miRs upregulated following EWS/Fli1 knock-down (miRs 22, 100, 125b, 221/222, 27a, and 29a), they showed that levels of these miRs vary specifically with EWS/Fli1 manipulation, including EWS/Fli1 depletion with different shRNAs and ectopic EWS/Fli1 expression in a heterologous fusion-negative cell line, and that these miRs are underexpressed in Ewing Sarcoma cell lines relative to MSCs. With respect to function, the authors demonstrated that forced expression of these miRs results in inhibition of growth in A673 cells, and that a subset of the miRs targets components of IGF signaling. These studies suggest that repression of select miRs promotes EWS/Fli1-driven oncogenesis by augmenting IGF pathway activity.

Franzetti et al. (2012) also profiled miRs in A673 cells following EWS/Fli1 depletion, but used an inducible shRNA approach and Illumina BeadChip technology as the profiling platform. These authors identified 34 upregulated, and 36 downregulated miRs upon EWS/Fli1 depletion. Focusing on miRs targeting CD99, they showed that miR-30a-5p, a miR downregulated by EWS/Fli1, contributes to the control of CD99 expression by EWS/Fli1. The authors further showed that overexpression of miR-30a-5p inhibits proliferation of and invasion by Ewing Sarcoma cells.

A number of studies have provided evidence in support of a MSC origin for Ewing Sarcoma (Castillero-Trejo et al., 2005; Riggi et al., 2005; Tirode et al., 2007). Thus, an alternative approach to identifying pathogenic miRs, including those driven by EWS/Fli1, in Ewing Sarcoma is comparison of miR expression profiles between Ewing Sarcoma and MSCs. Such an approach was taken by De Vito et al. (2011a). They identified 11 enriched and 24 depleted miRs in two Ewing Sarcoma cell lines (A673 and TC252) relative to MSCs. Among depleted miRs were multiple members of the let-7 family, previously shown to be tumor suppressive in other cancers. The authors showed that EWS/Fli1 directly represses let-7a expression, and that forced replacement of let-7a inhibits Ewing Sarcoma tumor xenograft growth, in part through regulation of HMGA2 levels.

Table 1 summarizes Ewing Sarcoma-associated miR alterations, as identified by the above studies. Several observations can be made. First, while some miRs (shown in bold) were identified in multiple studies, many miR changes were specific to a given study. Particularly striking are the differences between the EWS/Fli1-dependent expression profiles of Ban et al. (2011) versus those of McKinsey et al. (2011) and Franzetti et al. (2012) Several possible explanations exist. First, the use of transient EWS/Fli1 depletion by Ban et al. in contrast to the stable depletion used in the other two studies, may have enriched for miRs more immediately downstream of the fusion oncoprotein. Alternatively, the simultaneous comparison across multiple cell lines and tumors, as applied by Ban et al. may have imposed a very stringent filter that eliminated the detection of some miR alterations. Lastly, some of the differences may be accounted for by different controls and miR profiling platforms. Differences in profiling platforms may also be responsible for some of the differences between the miR alterations identified by McKinsey et al. and Franzetti et al. since both studies used the same cell line (A673), and same general approach (stable EWS/Fli1 knock-down). However, different controls, shRNAs, and culture conditions, as well as the precise depth of EWS/Fli1 knock-down, represent additional variables. Given the differences among studies using a similar experimental approach (EWS/Fli1 depletion), it is equally striking how similar the profiles are between the more divergent approaches taken by McKinsey et al. (depletion of EWS/Fli1) and De Vito et al. (2011a) (profiling of Ewing Sarcoma cell lines versus MSCs). The profile similarities between these studies suggest that, as observed with gene expression profiles (Tirode et al., 2007), depletion of EWS/Fli1 can give rise to a miR profile resembling MSCs. This would appear to provide further evidence that MSCs, or closely related cells, may be the cells of Ewing Sarcoma origin. Other noteworthy trends discernible from a cross-study comparison of miR alterations include the fairly consistent upregulation of members of the paralogous 17 ∼ 92a, 106b ∼ 25, and 106a ∼ 363 “oncomiR” clusters, and downregulation of miR-145. The biological consequences of most of the EWS/Fli1-related miR alterations identified in the above profiling studies await characterization.

**Table 1:** **Alterations in miR expression as a function of EWS/Fli1 or/and presumed cell of origin in Ewing sarcoma**.

	Ban et al. (2011)	McKinsey et al. (2011)	Franzetti et al. (2012)	De Vito et al. (2011a)
miRs up in EwS	500	**17/20a/106a/93**	34c	19a/19b
	126*	484	573	**17/20a/106a/106b/93**
	93*	**92a/92b/25**	**150**	**103/107**
	505	**15b**	486	**15b**
	128	**103/107**	**363**	18a
	126	324-5p	556	
	**9**	423-3p	9*	
	101	320	632	
	425*	let-7d	675	
	592	106b*	520a*	
	340*	130b	**106a**	
	505*	760	302b*	
	652	378	**9**	
	**150**	532-3p	346	
	20a*	665	1229	
		886-5p	504	
		574-5p	663	
		940	1270	
		296-3p	204	
		186	490	
		181d	**20b**	
			622	
			105	
miRs down in EwS	***145***	**146a/146b-5p**	452	*let-7* Family
	424	**21**	**145**	**199a-5p/199a-3p**
	**21**	***22***	144	**27a/27b**
	214*	***100****/99a/99b*	**143**	24
	214	***125b***	205	**193a**
	28-5p	***221****/222*	509	886-3p
	424*	584	190	**145**
	27a*	**199a-5p/199a-3p**	223	23a/23b
	22*	*29a*	**31**	**22**
	409-3p	***27a/27b***	767-3p	**143**
	21*	**193b**	**30a-3p**	**100**
	**125b**	549	511	**221/222**
	708	95	365	**31**
	135b	127-3p	517c	**125b**
		941	224	**21**
		203	450	
		574-3p	574	
		186*	668	
		493	**222**	
		922	34a	
		**30a**	**199b**	
		603	542-3p	
			137	
			***30a-5p***	
			**146a**	
			328	

Data from expression profiling studies using EWS/Fli1 depletion (McKinsey et al., 2011; Franzetti et al., 2012), Ewing Sarcoma versus mesenchymal stem cell comparison (De Vito et al., 2011a), or both (Ban et al., 2011). MiRs with shared seed sequence are grouped. MiRs in bold were detected as differentially expressed in at least two of the studies. MiRs in italics were shown to affect cancer phenotype(s) in Ewing Sarcoma. “*” Denotes the generally less abundantly expressed miR strand. See text and references for further details.

## MicroRNA Alterations in Tumor Cell Subpopulations

Tumors are inherently heterogeneous, and contain subpopulations of cells with unequal potential for tumor-initiation and maintenance. So-called cancer stem cells (CSCs) have been proposed to drive the initiation and maintenance or tumors, but defining their identity and impact remains a challenge for cancer biologists (Magee et al., 2012). Recently, a number of studies have explored the CSC concept in Ewing Sarcoma. Evidence has been presented in favor (Suva et al., 2009), and against (Jiang et al., 2010), CD133 expression as an important marker of Ewing Sarcoma cells with CSC-like properties, while another study identified ALDH expression as an alternative CSC marker (Awad et al., 2010). Expression of CD57 may be another phenotypic marker of Ewing Sarcoma cells with CSC-like properties (Wahl et al., 2010). MiRs have been widely implicated in the biology of normal stem cells and CSCs. To date, the role of miR biology as a function of CSC-like status in Ewing Sarcoma has been investigated in the CD133 model. One study, examining Ewing Sarcoma initiation in pediatric MSCs, identified miR-145 as an EWS/Fli1-repressed miR, and miR-145 repression, in turn, as a means to augment expression of EWS/Fli1 itself, as well as expression of the stemness-associated transcription factor Sox2 (Riggi et al., 2010). In subsequent work (De Vito et al., 2011b), the same group examined global miR expression differences between CD133+ and CD133− cell populations. Specifically, they compared the miR profiles of CD133+ and CD133− subpopulations from two Ewing Sarcoma tumors, pediatric MSCs stably expressing retrovirally introduced EWS/Fli1, and one Ewing Sarcoma cell line (STA-ET-8.2). The authors found more depleted than enriched miRs in CD133+ subpopulations, some of which were shared between Ewing Sarcoma tumors and EWS/Fli1-expressing MSCs, and identified downregulation of the miR processing factor TARBP2 as a mechanism for miR depletion in CD133+ cells. They further showed that TARBP2 depletion in Ewing Sarcoma cell lines results in downregulation of a group of miRs depleted in CD133+ cells and enhanced tumor xenograft growth, while augmentation of TARBP2 activity, via treatment with the fluoroquinolone-class antibiotic enoxacin, results in upregulation of these miRs and impaired tumor xenograft growth. Lastly, systemic administration of miR-143 or miR-145, two of the miRs downregulated in CD133+ cells, resulted in inhibition of tumor growth. Together, these findings support a role for miRs in the enhanced tumorigenicity of CD133+ Ewing Sarcoma cell subpopulations.

## MicroRNA Expression and Disease Prognosis

Nakatani et al. (2012) examined the potential role of miRs as predictive biomarkers in Ewing Sarcoma. In this investigation, global miR microarray profiling was performed on 34 primary Ewing Sarcoma tumors, comparing the expression profiles of patients with early relapse (median time from diagnosis 14 months, range 2–29 months) to those without clinical relapse (median follow-up 139 months, range 26–217 months). This analysis identified five miRs (34a, 23a, 92a, 490-3p, and 130b) that were significantly associated with both event-free and overall survival. In further analyses, low levels of miR-34a emerged as a particularly robust predictor of early relapse. In functional studies, the authors showed that miR-34a inhibits anchorage-independent growth of Ewing Sarcoma cell lines, and sensitizes to vincristine and doxorubicin. Consistent with the established role of p53 as a regulator of miR-34a expression, miR-34a levels were found to be low in Ewing Sarcoma cell lines with p53 inactivating mutations. Moreover, one tumor with low miR-34a levels was found to have a p53 mutation. This study examined p53 status in only a small number of tumors (six total). A question of interest for future studies is the extent of overlap between inactivation of the p53 pathway and miR-34a downregulation in Ewing Sarcoma tumors. On the one hand, miR-34a may emerge as a very useful surrogate marker of p53 pathway status, potentially independent of mechanism of p53 inactivation. Alternatively, miR-34a may identify a new subgroup of patients with poor prognosis.

## Mechanisms Responsible for Altered MicroRNA Expression in Ewing Sarcoma

What are the mechanisms responsible for the wide-ranging alterations in miR expression in Ewing Sarcoma? MiR biogenesis in the cell is regulated at multiple levels, including precursor (pri-miR) transcription (or transcription of the parent gene for miRs derived from protein-coding mRNAs), pri-miR to pre-miR processing, nuclear-cytoplasmic transport, pre-miR to (mature) miR processing, and stability/turnover. Thus far, in Ewing Sarcoma, evidence has been presented for regulation of precursor transcription and pre-miR to miR processing. With respect to transcriptional control, McKinsey et al. (2011) showed that a group of EWS/Fli1-repressed miRs also demonstrate downregulation of the precursor (pri-miR) transcript, consistent with a transcriptional regulatory mechanism. Further, three other studies of EWS/Fli1-repressed miRs [miRs 30a (Franzetti et al., 2012) and 145 (Riggi et al., 2010), and let-7a (De Vito et al., 2011a)] showed EWS/Fli1-dependent downregulation of the activity of the putative miR promoters in reporter assays. Most of the studies also examined binding of endogenous EWS/Fli1 to the presumed relevant miR upstream regulatory regions, using chromatin immunoprecipitation (ChIP). Interestingly, two studies found enrichment of EWS/Fli1 (De Vito et al., 2011a; McKinsey et al., 2011) at such regions, while two others did not (Ban et al., 2011; Franzetti et al., 2012). The tentative conclusion from these studies is that both direct and indirect mechanisms appear to contribute to transcriptional miR repression by EWS/Fli1, with detailed mechanisms awaiting further elucidation. Indirect mechanisms could involve the action of EWS/Fli1-induced repressors, like Nkx2.2 (Owen et al., 2008), NR0B1 (Kinsey et al., 2006), and EZH2 (Richter et al., 2009). Alternatively, EWS/Fli1 could repress miR transcriptional activators. Thus far, studies of transcriptional miR regulation in Ewing Sarcoma have only addressed downregulated miRs. Potential mechanisms of transcriptional activation of miR expression by EWS/Fli1 remain to be examined.

**Figure 1:**
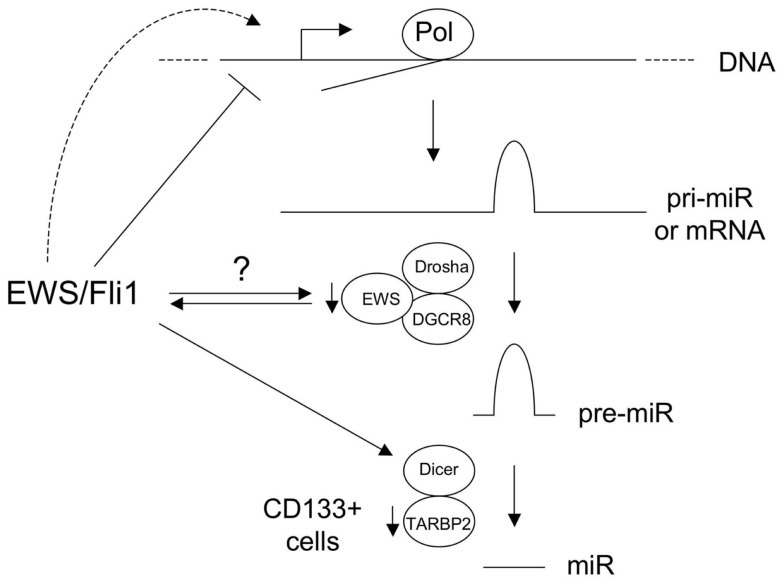
**Known and hypothetical mechanisms of altered microRNA (miR) expression in Ewing sarcoma**. Basic pathway for biogenesis of majority of miRs is shown, including those derived from non-coding RNA precursor transcripts (pri-miR) and protein-coding mRNAs. EWS/Fli1 represses some miRs at the transcriptional level, through direct and indirect mechanisms, and likely also transcriptionally activates other miRs. EWS is a component of the Drosha/DGCR8 miR processing complex, and the copy number of the wild-type intact form is reduced in Ewing sarcomas with EWS-containing fusions. EWS/Fli1 can also interact with EWS. The consequences of this to miR biogenesis are currently unknown. Dicer is upregulated by EWS/Fli1; the consequences of this in Ewing Sarcoma are unknown, but Dicer levels impact oncogenesis in other cancers. TARBP2 downregulation in CD133+ Ewing Sarcoma cells results in diminished expression of a number of miRs. (Pol: RNA Polymerase.) See text and references for more detail.

Studies have also provided evidence for alterations in miR processing in Ewing Sarcoma. De Vito et al. (2011a) identified upregulation of Lin-28B and hnRNPA1, both inhibitors of let-7 maturation. As discussed above, in a subsequent study, the same group also showed impaired miR maturation due to diminished levels of TARBP2 in the CD133+ subpopulation of Ewing Sarcoma cells (De Vito et al., 2011b). The mechanisms of Lin-28B and hnRNPA1 upregulation, as well as CD133+ subpopulation-specific TARBP2 downregulation, remain to be determined. An intriguing, and currently largely unexplored, possibility is a role for the unrearranged copy of EWS or/and the EWS component of EWS/Ets fusions, in miR processing in Ewing Sarcoma. EWS has been identified in the (pri to pre) miR processing complex (Gregory et al., 2004), and a recent study suggests that it can promote the processing of some miR(s) (Sohn et al., 2012). Roles in the promotion of miR processing have also recently been demonstrated for the other two members of the TET (TLS/EWS/TAF15) family, FUS/TLS (Morlando et al., 2012), and TAF15 (Ballarino et al., 2012). Whether EWS has a more general role in miR processing, whether potential haploinsufficiency for this function affects miR biogenesis in Ewing Sarcoma, and whether EWS/Ets fusions interfere with this function, through EWS/Ets-EWS protein-protein interactions (Embree et al., 2009) or through gain-of-function miR processing activity of EWS/Ets, remain to be determined. Lastly, Dicer, a key effector of miR maturation in the cell, is an EWS/Fli1-upregulated gene (Kinsey et al., 2006). The functional consequences of this to miR regulation and disease phenotype in Ewing Sarcoma are currently unknown, but changes in Dicer levels can profoundly influence cancer phenotypes (Lambertz et al., 2010; Nittner et al., 2012). The above mechanisms are summarized in Figure 1.

## Conclusion and Perspectives

What have we learned from miR studies in Ewing Sarcoma thus far? We have gained new insight into disease biology, and identified new candidate biomarkers and therapeutic targets. MiRs represent a novel and unique mechanism of regulation of gene expression, which can affect protein levels without dramatic changes in mRNA levels. Thus, understanding of miR-mediated pathways can reveal new oncogenic mechanisms not easily accessible to other methodologies like RNA-based gene expression profiling. To this end, miR studies thus far have revealed a new mechanism for augmenting EWS/Fli1 protein levels (Riggi et al., 2010; Ban et al., 2011), new mechanisms of induction of known upregulated genes (CD99) and pathways (IGF) in Ewing Sarcoma (McKinsey et al., 2011; Franzetti et al., 2012), and new players in the EWS/Fli1-driven oncogenic program (HMGA2 and Sox2) (Riggi et al., 2010; De Vito et al., 2011a). Of clinical relevance, the work of Nakatani et al. has identified a potential miR-based biomarker of aggressive disease (miR-34) (Nakatani et al., 2012), which could have real clinical utility given the relative ease of assaying miR levels in tumor specimens (Hui et al., 2009). Preclinical animal studies have also suggested potential candidate strategies for miR-based therapeutics, including replacement of miR-145 (Riggi et al., 2010; De Vito et al., 2011b), miR-143 (De Vito et al., 2011b) and let-7a (De Vito et al., 2011a), and augmentation of TARBP2 activity (De Vito et al., 2011b). Many interesting unanswered questions remain. A great deal remains to be learned about the mechanisms responsible for altered miR expression in Ewing Sarcoma. Similarly, the biology of individual miRs with altered expression patterns remains largely uncharacterized. Particularly intriguing is the role of miRs like miR-21 and miRs-221/222, identified as upregulated and pro-oncogenic in most malignancies (Sotiropoulou et al., 2009; Visone and Croce, 2009), but observed to be downregulated in most profiling analyses of Ewing Sarcoma (see Table 1). Further, to date, studies of miR biology in Ewing Sarcoma have been carried out in the context of the common EWS/Fli1 fusion. It will be of interest to determine how miR expression and function differ in the context of the other, less common, EWS/Ets fusions, as well as the more divergent non-EWS/Ets fusions discovered recently (Sankar and Lessnick, 2011). Will miR-based therapies make it to the clinic? As with all new concepts and methodologies, only time and more rigorous science will tell.

## Conflict of Interest Statement

The authors declare that the research was conducted in the absence of any commercial or financial relationships that could be construed as a potential conflict of interest.
